# Editorial: The microbiology of the biogas process

**DOI:** 10.3389/fmicb.2023.1235624

**Published:** 2023-06-20

**Authors:** Ana J. Cavaleiro, Joana I. Alves, Andreia F. Salvador, Alfons J. M. Stams, M. Alcina Pereira, M. Madalena Alves

**Affiliations:** ^1^CEB—Centre of Biological Engineering, University of Minho, Braga, Portugal; ^2^LABBELS—Associate Laboratory, Braga, Portugal; ^3^Laboratory of Microbiology, Wageningen University and Research, Wageningen, Netherlands

**Keywords:** biogas/biomethane, anaerobic digestion, microbial communities, microbial diversity, microbial interactions, waste/wastewater, sustainability

The world is facing unprecedent challenges, related with energy crisis and climate change. Intensification of renewable energy production, with special focus on sustainable biogas and biomethane, is one of the front-line topics today. Biogas/biomethane will play a role in the transition toward climate-neutral and secure energy system. Ensuring that biomethane is produced from organic waste/wastewater is essential to support circularity and sustainability. Scaling up biomethane production and assuring its economic competitiveness are current key challenges.

Biogas generation occurs in natural and engineered environments, such as anaerobic bioreactors, and involves a cascade of reactions catalyzed by complex microbial communities. To unlock and boost the full potential of waste-based biomethane production, bioprocess optimization is needed, which requires deep knowledge on microbial diversity and physiology, as well as on the complex microbial interactions and metabolic networks occurring in biogas processes. This was the motivation for launching the Research Topic “*The Microbiology of the Biogas Process*,” which comprises six original research articles by 48 authors, addressing different facets of the theme and resorting to diverse approaches, reflecting the complexity of the topic.

Expanding the range of biomass feedstocks for biomethane production is currently a priority. Special focus has been placed on lignocellulosic agricultural residues, but their recalcitrant structure causes considerable challenges to microbial degradation. Improving the hydrolysis step will reinforce the exploration of these raw materials for sustainable biomethane production. Jensen et al. studied the microbial communities involved in the degradation of lignocellulosic-rich wheat straw in continuous stirred tank reactors. Cellulase and xylanase activity profiles were correlated with changes in microbial community composition. The results show the occurrence of reactor-specific adaption of the different (mesophilic and thermophilic) inocula, and representatives of *Ruminiclostridium, Caldicoprobacter, Ruminofilibacter, Ruminococcaceae, Treponema* and *Clostridia* groups were identified as key species in lignocellulose degradation. The correlation method that was developed is a promising approach for the identification of potential cellulolytic and xylanolytic microorganisms in the anaerobic digestion (AD) of lignocellulosic biomass.

During lignocellulose degradation, aromatic compounds such as phenyl acids (PA) can accumulate in AD, with effects that are not fully understood yet. In the research paper of Prem et al. the effect of three different PA, alone or in mixture, was studied in thermophilic AD. Different substrates were tested, targeting various AD stages/microorganisms – (i) microcrystalline cellulose (MC), involving all the typical AD microbial groups; (ii) butyrate and propionate to study the syntrophic relation of acetogens with methanogens, and (iii) acetate, focused on syntrophic acetate oxidizers and/or acetoclastic methanogens. In the presence of PA, methane production decreased, and this effect was more pronounced in the assays with VFA (up to 93%), and lower (9–24%) for MC. A higher quantity of extracellular polymer substances (EPS) was produced in the MC assays, pointing that EPS were working as protective agents against PA toxicity. Moreover, acetoclastic methanogenesis prevailed in the assays with propionate and butyrate without PA, but was inhibited in the presence of PA.

Developing and testing sustainable and cost-effective upstream and mainstream strategies is important to improve the conversion of more recalcitrant organic wastes into energy. Saba et al. successfully enhanced biogas production from chicken feather (CF) by pretreatment with a keratinase-secreting *Pseudomonas aeruginosa* and co-digestion with rice husk or green grocery waste (GW). The pretreatment of CF was applied to disrupt their recalcitrant structure and promote hydrolysis, while co-digestion aimed to reduce ammonia inhibition. CF pretreatment followed by batch co-digestion with rice husk increased 34% the biogas yield. Continuous AD of pretreated CF and GW enhanced process stability and reduced ammonia toxicity, resulting in higher biogas production, relatively to the mono-digestion of pretreated CF.

Besides biogas, medium chain carboxylates (MCC) can be produced by open cultures in bioreactors, presenting high economic value and wide applications in the chemical industry. Bioreactors' operation at mildly acidic pH values facilitates the extraction of MCC, but only few bacteria have been isolated that are capable of producing MCC at these pH values. Esquivel-Elizondo et al. isolated a chain-elongating bacterium that grows at mildly acidic pH and mostly produces *n*-caproate from carbohydrates. The isolate is assigned to the *Caproiciproducens* genus. The authors examined the whole genome of the isolate, and studied the reverse β-oxidation (rBOX) genes to get further insight of its metabolism. Potential application of this novel bacterium in bioreactors that are aimed at MCC production from organic wastes is also discussed.

Bioaugmentation is another strategy that has been frequently applied to improve the performance of AD systems, but its outcome is not always easy to predict. To elucidate the bioaugmentation mechanisms of anaerobic granules, Doloman et al. developed a computational model that visually demonstrates the spatial stratification of the different bacterial and archaeal groups, as well as the distribution of the metabolic products. Bioaugmentation of cellobiose-degrading granules with oleate-degrading bacteria, to mimic the shift to a lipid-rich feed, was chosen as case study, and the results visually demonstrate the importance of the substrate-specific niche and impact of washout on the bioaugmentation outcome. The proposed model is a useful tool to predict the effects of bioaugmenting anaerobic granules in case of a change to a new feed type.

Microbial syntrophy has a key role in the anaerobic breakdown of organic compounds to methane. The main enzymes involved in fatty acids degradation though β-oxidation have been identified, but questions still remain about substrate specificity and regulation of paralog genes. Fu et al. used mass spectrometry-based proteomics to characterize and compare the acylome profiles of two *Syntrophomonas wolfei* subspecies grown on different carbon sources. Extensive changes were detected in acylation-type, abundance, and modification sites, pointing that protein acylation by reactive acyl-Coenzyme A species (RACS), generated during fatty acids metabolism, may be an important post-translational regulatory mechanism of syntrophy.

In summary, this Research Topic explores both applied and fundamental aspects of anaerobic microbial communities thriving in biogas processes, highlighting the importance of key microbial players and crucial microbial interactions on the performance and stability of these systems. We hope that this Research Topic will inspire future works pursuing the goals of sustainable waste-based high-performance bioenergy-producing AD systems.

## Author contributions

AC and JA main activities for drafting of the editorial. ASa, ASt, MP, and MA revision of draft. All authors contributed to the article and approved the submitted version.

